# Bonded Green Exercise: A One Health Framework for Shared Nature-Based Physical Activity in the Human–Dog Dyad

**DOI:** 10.3390/ani16020291

**Published:** 2026-01-16

**Authors:** Krista B. Halling, Mark Bowden, Jules Pretty, Jennifer Ogeer

**Affiliations:** 1 KH Veterinary, Burlington, ON L7P 0A6, Canada; 2 TRUTHPLANE, Toronto, ON M5R 1R6, Canada; mark@truthplane.com; 3 School of Life Sciences, University of Essex, Colchester CO4 3SQ, UK; jpretty@essex.ac.uk; 4 Centre for Comparative Medicine, University of British Columbia, Vancouver, BC V6S 0K5, Canada; jogeer@mail.ubc.ca

**Keywords:** bonded green exercise, canine science, human–animal bond (HAB), human–animal interaction (HAI), human–dog dyad, human–nonhuman animal relationship, One Health, public health, social behaviour in mammals

## Abstract

Modern lifestyles are increasingly plagued by physical inactivity, social disconnection, digital addiction, and excessive time indoors—factors that negatively impact the health and well-being of both humans and their companion dogs (*Canis familiaris*). Evidence shows that nature exposure, physical activity, and human–animal bond (HAB) each enhance physical, mental, and social well-being, yet these domains have rarely been examined together as an integrated therapeutic triad. We introduce a new conceptual framework of bonded green exercise, defined as shared physical activity between a bonded human and dog in natural environments. Synthesizing existing evidence across human and canine sciences into a testable conceptual integration, we posit that bonded green exercise may plausibly activate evolutionarily conserved, synergistic mechanisms of physiological, behavioural, and affective co-regulation. Four testable hypotheses are proposed: (H1) triadic synergy: combined domains produce greater benefits than additive effects; (H2) heterospecific benefit: parallel health gains occur in both species; (H3) behavioural amplification: dogs acts as catalysts to drive human participation in nature-based activity; and (H4) scalable health promotion: bonded green exercise represents a low-cost, accessible, One Health approach with population-level potential. This framework highlights how intentional, shared physical activity in nature may potentially offer a novel low-cost and accessible model for enhancing health, lifespan, welfare, and ecological stewardship across species.

## Simple Summary

Modern adopted lifestyles tend to keep humans and their dogs predominantly indoors, inactive, and disconnected from nature. This contributes to both species stress, loneliness, and declining physical health. While nature exposure, physical activity, and the human–dog bond are each known to support well-being, they have rarely been examined together. In this paper, we introduce the testable concept of “bonded green exercise”—humans and their dogs being active together in natural outdoor settings. We suggest that experiencing these three elements (nature exposure, physical activity, and the human–animal bond) together may plausibly benefit both species by reducing stress, improving mental and physical health, and strengthening their bond. Dogs may plausibly also motivate humans to be more active, creating a positive cycle that further supports well-being for both partners. By defining this new concept and explaining why bonded green exercise may plausibly offer health benefits to both dogs and humans, we aim to guide future research and highlight a simple, accessible behaviour that may potentially improve well-being and welfare for humans, dogs, and communities.

## 1. Introduction

Today’s hyper-convenient lifestyles are paradoxically plagued by the costs of digital addiction [1,2], sedentary behaviour [3], social disconnection [4], and a disproportionate time spent indoors [5,6,7], affecting not only people but also the dogs (*Canis familiaris*) [8,9], who share the lives and environments of humans. We are living through simultaneous epidemics of loneliness [4,10], digital addiction [1]; mental health struggles [11], physical inactivity [12,13], and obesity [14], which, through shared anthropogenic environments and lifestyles, collectively affect both humans [11,15] and pet dogs [5,11] worldwide. As stated soberly by Barton et al. [13] (p. 23), “Never in human history have humans, as a species, moved bodies so far with so little physical effort.” Amid rising rates of chronic, debilitating and non-communicable diseases, emotional dysregulation, and social isolation across these species, the need for low-cost, accessible strategies that promote physical and emotional well-being in both humans and dogs is timely and urgent.

Ways of living are increasingly characterized by indoor and urban living. In North America and Europe, people spend approximately 90% of their time indoors with only about 6% spent outside [6,7,16,17]. Nearly one-third of Canadians report not spending any time outside on a typical day, and among those who do, the average duration is under an hour [6]. These figures are not entirely surprising, given the sharp rise in urbanization. Globally, 55% of the population currently lives in urban areas, a figure projected to rise to 68% by 2050 [18]. These shifts have profound implications for physical activity, access to green space, and social connection. The 2025 Position Statement on Active Outdoor Play [12] (p. 1) states “Active outdoor play promotes holistic health and well-being for people of all ages, communities, and environments, and for our entire planet. It is critical given the multiple global challenges we face today”.

Humans form layered relationships with animals—from ecological and working partnerships, to domestic companionships, to the deeply integrated bond shared with dogs. Throughout human history, relationships with animals have played a vital ecological, cultural and emotional role [19]. Our fascination with animals is reflected in their common appearance in ancient rock art [20], and as protagonists in story and myth [21]. In our daily lives, humans have long relied on animals for sustenance, labour, protection, and companionship, and these multi-species relationships have shaped both our evolution [11] and our psychology [22]). From horses kept for transportation, sport or companionship, to livestock that sustain our survival, to pets who share our homes as companions, our interactions with animals reflect a wide interdependence.

One species in particular stands out–*Canis familiaris*, the domestic dog. Today there are an estimated 470 million pet dogs worldwide, including nearly 90 million in the United States (where 46% of households own a dog) [23], 10 million in Spain [24], 8 million in Canada [25], and 13 million in the United Kingdom where 31% of households own a dog [26]. Many dogs are considered by their owners to be a member of the family [27,28,29]. Recently, jurisdictions such as British Columbia and New York City have legally recognized pet dogs as family members rather than possessions. Similarly, dog ownership has been equated with an increase in life satisfaction equivalent to that derived from earning an additional £70,000 per year [30]. 

Humans and dogs sharing lives and lifestyles is not a recent development. Dogs were the first domesticated species, sharing some 15,000–30,000 years of evolutionary history with humans [31], and today remain uniquely and emotionally integrated into our lives. Like all human–animal partnerships, this relationship carries cultural and ethical complexities [11]. However, its depth of attunement and co-regulation remains unparalleled. Dogs’ ability to form strong attachment bonds, synchronize behaviour, and share living environments with humans sets them apart from all other domestic species [15,32]. This enduring and ubiquitous human–dog partnership provides a distinctive model for exploring how interspecies relationships influence health and well-being [11,33].

Indeed, the costs of modern lifestyle are not confined to humans. Pet dogs, who share human homes and routines, mirror this sedentary, indoor existence and its associated health consequences [34]. Rising rates of canine obesity [14], orthopedic disease, cancer [34], allergy [35], and behavioural stress [36] have paralleled human trends, underscoring that dogs are not neutral bystanders but affected members of a shared psychosocial environment and this modern crisis. These outcomes are not only physical but psychological [11,36].

As Martin et al. [22] describe, in both humans and dogs, healthy psychological function requires autonomy, competence, and relatedness. When these core needs are unmet, such as being in an understimulated and unenriched environment, maladaptive outcomes may arise. As dogs live in human-managed environments, their ability to exercise choice (autonomy), feel capable and effective (competence), and regulate their emotional and attachment level (relatedness) depends almost entirely on their human caregivers [22,37]. This dependence makes dogs vulnerable to the same effects of a sedentary, indoor lifestyle that undermines human well-being. Similarly, this shared susceptibility and shared environment make dogs a particularly relevant and responsive partner species through which to explore accessible, health-promoting interventions at the individual and population level [34].

This human–animal vulnerability aligns with the concept of One Health, which, as defined by the World Health Organization (WHO), “is an integrated, unifying approach that aims to sustainably balance and optimize the health of people, animals and ecosystems” [38] (paragraph 1). Importantly, global leaders—including WHO, the Food and Agriculture Organization of the United Nations, the United Nations Environment Programme, and the World Organization for Animal Health (WOAH)—have recently stated the urgency of advancing One Health as essential for sustainably addressing health risks at the human–animal–environment interface [39].

In light of the above, and in the face of today’s multitude of global lifestyle crises, a substantial body of existing and emerging evidence supports the positive human and/or canine health outcomes associated with each of the following three key domains: (1) nature exposure, (2) physical activity, and (3) the human–animal bond (HAB).

Nature exposure improves many aspects of health, including stress regulation, cognition, sleep, mental health, and immune function [40]. Physical activity enhances cardiovascular, metabolic, and emotional health, as well as body condition score [41]. The human–animal bond confers stress regulation [32,42,43,44,45], social buffering [32,46], co-regulatory benefits [15,32,33,44,45,47], and increased motivation to exercise [48,49,50], including a positive correlation between strength of the attachment bond and the distance owners walk their dogs [51]. 

Furthermore, as noted by Pretty [52], humans form multi-layered relationships with the world around them, ranging from whole-place attachments to ecosystems, landscapes, and multispecies communities, to individuated emotional bonds with other beings, including dogs. Just as community-level connection and social capital have well-documented health benefits for humans, so do close interpersonal relationships. The HAB, in this light, represents an interspecific form of “togetherness”, a construct often aligned with social capital [52]. Such multi-level togetherness, from communities to cross-species bonds, has been linked to increased life satisfaction and longevity through mechanisms of social capital and mutual care [52,53,54].

Despite the wealth of research on these three individual domains, they have rarely been studied as an integrated triad [55]. While paired combinations, such as nature + exercise (coined green exercise by Pretty et al. [56]), have received extensive attention [49,57,58] and demonstrate synergistic benefits [56,59], a surprising gap remains in understanding what occurs when all three are experienced simultaneously by a human and their dog. Yet this is precisely what occurs when people and their dogs engage in shared outdoor activities such as walking, hiking, or adventuring together–a behaviour that is free, accessible, and enjoyed by many people worldwide.

Recent studies [55,60] have begun to explore shared outdoor exercise between humans and dogs, and note that this research gap exists. To our knowledge, no conceptual model has yet been proposed to examine the full triadic interaction of nature exposure, physical activity, and the human–animal bond within a human–dog dyad. Bonded green exercise is therefore proposed as a hypothesis-generating framework that integrates three well-established domains into a single, testable, One Health concept to guide empirical studies.

Here we present a conceptualized and testable model for intentional shared activity in nature as the primary unit through which physical, environmental, and relational processes may co-occur and may plausibly shape one another. Treating the shared dyadic activity itself as a coherent behavioural system allows bonded green exercise to be explored as a distinct cross-species context with hypothesized implications for motivation, adherence, emotional regulation, and well-being.

Large-scale nature-based and mind–body interventions [61] have already demonstrated measurable public-health benefits and substantial cost savings. Given the global prevalence of dog ownership and the ubiquity of public green spaces, a comparable intervention incorporating human–dog activity in nature would be predicted to plausibly confer similar population-level health and economic returns.

### 1.1. Framework Overview

In this paper, we propose the novel concept of bonded green exercise to describe this triadic interaction: shared physical activity in nature between a human and a dog. This framework builds on the well-established concept of green exercise by explicitly integrating the human–animal bond as a third, co-active domain. Bonded green exercise therefore treats shared human–dog activity in natural settings as a single integrated behavioural context, rather than as outcomes distributed across adjacent domains. Importantly, we present this framework as a provisional model to guide future studies, rather than as an empirical conclusion.

#### Synergistic Effects

Based on this conceptual framework, bonded green exercise is posited to, at a minimum, potentially confer meaningful additive health outcomes to both species, associated with all three domains. Moreover, we hypothesize that the simultaneous engagement of the domain triad may plausibly yield benefits that are synergistic and co-regulatory.

In both biological and behavioural systems, synergistic interactions may arise when two or more factors act in concert, through interactive or mutually reinforcing processes (such as physiological or behavioural positive feedback loops), to produce an amplified effect compared to the sum of effects of the individual factors [62,63]. In the context of bonded green exercise, we posit that synergistic effects may plausibly emerge through shared physiological pathways and/or through behavioural reinforcement, whereby engagement in one domain may plausibly enhance the intensity, duration, or quality of engagement in the others.

While evolutionary theory [11,42,64] lends biological plausibility for this synergistic potential, its hypothesized expression in bonded green exercise contexts remains an empirical question. Humans and dogs co-evolved over tens of thousands of years as cooperative partners in physically active tasks within natural environments, including hunting, herding, gathering, and migration [31,64,65,66,67,68]. This long history of shared activities and cohabitation is believed to have shaped dogs not only by convergent evolution, but also through the domestication process [11], jointly selecting for the uniquely interspecific attachment bond recently identified between dogs and humans [69,70].

These same evolutionary pressures are thought to have likely also favoured cross-species synchrony of emotions [71,72,73], behaviour [66,72,74,75,76,77], and physiology [45,78,79,80], allowing dogs and humans to co-regulate in alignment with shared tasks and environments [15,33,47,64]. For example, the reciprocal oxytocin (OT) feedback loop between humans and dogs facilitates powerful interspecific stress co-regulation [45], potentially offering emotional buffering that is especially meaningful amid widespread mental health challenges.

Yet while evolutionary theory offers compelling hypotheses on the foundations of these interspecies dynamics, we argue that bonded green exercise is not a genetically predetermined behaviour. We propose that evolution plausibly provided the template for this deep connection, and hypothesize that bonded green exercise may be an accessible way to intentionally activate this pathway in a modern context. Human–dog partnerships are shaped by both biology and culture; emergent from choices, relationships, and environmental affordances. As Graeber and Wengrow [81] emphasize, human systems evolve through agency as much as through adaptation. Framing bonded green exercise as an intentional lifestyle shift opens space for proactive public health interventions.

As such, we argue that these processes are shaped not only by evolutionary inheritance but also by human and canine choice within social and cultural contexts. While the biophilia hypothesis [42,82] proposes an innate human tendency to affiliate with nature and animals, this should be viewed not as a deterministic effect, but as an evolved potential and one that is modulated by context, choice, and culture [52].

### 1.2. Our Hypotheses

Bonded green exercise may plausibly represent a uniquely potent, low-cost, and biologically familiar behaviour. By integrating shared movement in a natural setting, we posit that bonded green exercise may plausibly provide a prime context for activating sensorimotor synchrony, emotional connection, and evolutionarily preserved pathways of well-being in both humans and dogs. As such, we propose the following testable hypotheses:

**Triadic** **synergy** **hypothesis** **(H1).** *That simultaneous engagement of nature exposure, physical activity, and HAB will produce greater health benefits for both humans and dogs than the additive benefits of the domains experienced individually, due to potential interaction among shared regulatory and motivational systems.*

**Heterospecific** **benefit** **hypothesis** **(H2).** *That bonded green exercise yields physical and emotional health benefits in both humans and dogs, consistent with the goals of One Health.*

**Behavioural** **amplification** **hypothesis** **(H3).** *That dogs may act as catalysts for increased human engagement in physical activity in nature, thereby increasing the motivation and frequency of green exercise participation by humans.*

**Scalable** **health** **promotion** **hypothesis** **(H4).** *That given its low cost and accessible nature, bonded green exercise may represent a scalable public-health strategy to improve population-level mental and physical health in both species.*

This novel framework bridges systemic and individual forms of connection, linking the ecosystem-level benefits of green exercise with the interpersonal benefits of the human–animal bond. Bonded green exercise further imparts relevance for multiple disciplines, including canine science [83], veterinary behavioural health, public health, HAB and HAI science, exercise physiology, evolutionary biology, environmental psychology, and One Health.

By naming and defining bonded green exercise and outlining testable hypotheses, we invite scientific investigation into an existing yet underexplored dyadic behaviour, with the plausible potential to enhance physical, mental, and emotional health across species.

## 2. Definitions and Conceptual Landscape

### 2.1. Green Exercise

Green exercise has been defined as physical activity conducted in natural environments, with a growing body of evidence on its mental, physical, and social benefits in humans [56,58,59,84]. Spending time in nature—whether on land or on water—is also referred to as green time or blue time, respectively.

### 2.2. Bonded Green Exercise

Bonded green exercise, as introduced here, extends the construct of green exercise by explicitly incorporating a third domain: the human–animal bond (HAB). We define bonded green exercise as human–dog shared physical activity in nature, thereby simultaneously exposing a human and their dog to physical activity, nature, and the human–animal bond. This definition motivates our central hypotheses (H1–H4): that full triadic co-activation plausibly yields synergistic effects (H1), confers parallel benefits across species (H2), is catalyzed by dogs’ motivational effects on human outdoor physical activity (H3), and is scalable as a public-health strategy (H4).

### 2.3. Shared Physical Activity

It is important to note that shared physical activity, in the context of bonded green exercise, refers to intentional, co-active movement in which both the human and the dog are engaged in a common purpose, with the human remaining attentive and responsive to the canine partner. This definition emphasizes the quality of mutual engagement rather than merely simultaneously being physically active. For instance, walking one’s dog while being distracted (such as scrolling on a mobile device) may provide physical activity but not relational attunement central to the bonded green exercise framework. Thus shared physical activity implies a mindset of presence and cooperation, where the human regards the activity as a means of cooperative enjoyment with their canine rather than a task or an obligation.

Recognizing that the human–animal bond exists along a continuum, and the strength of the attachment bond correlates with co-regulatory and motivational mechanisms central to bonded green exercise, this framework focuses primarily on human–dog dyads who have ongoing interaction and social familiarity.

### 2.4. Human–Animal Interaction and Human–Animal Bond

While much of the literature on humans and dogs is framed as human–animal interaction (HAI), this is a broad term encompassing any context in which humans and animals engage or coexist. Within this umbrella, animal-assisted interventions (AAI)—including animal-assisted therapy, education, and activities—represent structured, goal-oriented programmes that harness the human–animal relationship to improve physical, emotional, or cognitive health outcomes in people. Similarly, the role of working and service dogs provides compelling evidence that dogs can deliver measurable physiological and psychological benefits through partnership and shared activity [85,86].

While we posit that bonded green exercise shares similar mechanisms, such as physiological co-regulation, emotional buffering, and social motivation, it differs in context and intent. Rather than being prescribed or human-centric, bonded green exercise may occur naturally within everyday life and emphasizes reciprocal benefit for both members of the dyad. Accordingly, in this paper we focus on the human–animal bond (HAB) as the central mechanism of interest. Whereas HAI denotes concurrent presence or interaction, HAB in this context refers specifically to the attachment-based, reciprocal, and enduring qualities of the human–dog relationship.

### 2.5. Existing Constructs

Existing constructs in the scientific literature capture partial domain overlaps. For example, green exercise focuses on humans being active in nature but omits the relational dimension, while dog walking is often studied as a utilitarian behaviour aimed at fitness or canine needs rather than as a co-active, emotionally reinforcing practice. Bonded green exercise differs by emphasizing the relational mechanisms of attachment, co-regulation, and synchrony that may arise in human–dog dyads, in addition to both physical exercise and exposure to nature.

These properties and definitions are essential for understanding the hypothesized synergistic impact of bonded green exercise in the following framework.

## 3. Theoretical Framework and Evolutionary Foundations

### 3.1. Why the Combination of These Domains Matters

The health benefits of nature exposure, physical activity, and the human–animal bond are well established when studied independently. Green exercise has demonstrated in humans synergistic benefits of physical activity and nature. To the authors’ knowledge all three domains have not explicitly been studied in unison, yet when people and their companion dogs move together in natural environments, all three domains might be activated at once. As each domain independently confers established health benefits, their simultaneous exposure within the dyad has the potential to yield, at a minimum, additive health outcomes.

We hypothesize (H1) that this triadic activation may produce effects greater than the sum of individual domains. This hypothesis is informed by evidence (as noted in Section 5) that paired domain combinations may yield synergistic effects, and on the conceptual premise that simultaneous engagement of multiple domains increases the plausibility of interaction of underlying pathways extending beyond simple additivity. We posit that shared outdoor activity may further act as a re-activator of latent mechanisms such as attunement, stress regulation, and trust—processes that are recognized to be most readily expressed in dynamic, multisensory environments [43,53,64]. Novelty in natural settings may be especially important, heightening attention, curiosity, and engagement for both partners, and plausibly creating conditions for deeper presence, trust, and bonding [46,53,87].

Since both the human and dog are engaged in these conditions simultaneously, we further hypothesize (H2) that the resulting benefits may be mutual in both species.

### 3.2. Hypothesized Evolutionary Basis for Bonded Green Exercise

Evolutionary history lends plausible biological support to our bonded green exercise hypotheses. Over tens of thousands of years, humans and dogs co-evolved through cooperative activity in natural environments, including hunting, guarding, foraging, herding, and migration [31,64,66]. These tasks, under natural and artificial selection pressure for survival and co-existence in humans and dogs, allowed for optimized mutual traits such as responsiveness to each other’s nonverbal cues, co-operative problem-solving, exploratory drive, as well as deep trust and social bonding [88]. This long-standing reliance on nonverbal signalling is consistent with broader accounts of how synchrony and embodied cues shape trust and interaction across social mammals (e.g., [66,89]).

Natural and artificial selection have been thought to favour human–dog dyads that were highly coordinated and socially attuned, as this connection likely afforded mutual survival and reproductive advantages by improving their ability to acquire food and fend off threats [68,90]. Indeed, evidence on dog domestication supports that dogs were selectively favoured for affiliative and cooperative behaviours that enhanced their fitness in anthropogenic environments [66,78,91,92]. 

The domestication hypothesis suggests that dogs were further shaped for attentiveness to human signals and relational compatibility, with dogs outperforming even primates in their sensitivity to human gaze and gesture [66,78]. These convergent adaptations, combined with human capacities for attachment and care, produced a unique heterospecific partnership [32,69,70].

In contrast to the tens of thousands of years during which human–dog dyads thrived through shared outdoor activity, today’s sedentary indoor lifestyle represents a mere blip in their parallel evolutionary timeline—and stands in stark opposition to the context in which these biological systems have been optimized. This modern environmental mismatch [93] may, at least in part, underlie both the health costs of sedentary indoor living and, conversely, the restorative effects of physical activity and nature exposure.

To that end, we posit that shared activity in green environments in modern contexts may plausibly provide more than incidental exercise or enrichment. We hypothesize that bonded green exercise may plausibly mitigate the biological discordance of today’s lifestyle (see [93]) by plausibly re-engaging in human–dog dyads these co-evolved systems for coordination, communication, and autonomic regulation. This hypothesized and provisional evolutionary framing aligns with recent conceptual work positing the human–dog dyad as a co-evolved biologically integrated regulatory system [33].

The contextual overlap of bonded green exercise with that of ancient times lends plausibility to our hypotheses (H1 and H2) that bonded green exercise may be not simply serve as a lifestyle choice but, we further hypothesize, that it may plausibly also function to re-activate ancient, conserved and biologically optimized pathways that promote health, resilience, and relational strength in both species.

### 3.3. Why Bonded Green Exercise Matters Now

As aforementioned, today, many humans and dogs are embedded in predominantly indoor, urban contexts. Over half of the global population now lives in cities, a proportion expected to rise sharply in coming decades, opportunities for connection and outdoor exercise are increasingly scarce yet equally vital [12]. The rise in urbanization and related lifestyle shift have contributed to digital addiction in humans and simultaneous epidemics of obesity, chronic disease, and mental health challenges collectively affecting both species [1,11,14].

Moreover, recent evolutionary analyses by Longman and Shaw [93] warn that many humans are now living in profound “environmental mismatch,” where rapid post-industrial lifestyle changes—particularly sedentary, indoor, and screen-dominated behaviours—are increasingly misaligned with the movement-rich, socially embedded natural environments in which our physiology evolved.

Time spent outdoors has decreased to the point that many physicians are prescribing “time in nature” as a remedy for physical and mental health ailments [40]. In the scientific literature, such green time is also referred to as “nature-based intervention” [61].

The 2025 Position Statement on Active Outdoor Play [12] was recently released by an international leadership group in response to the increasing global prevalence of digital devices and the resulting shift to indoor, sedentary activities. They highlight the widespread physical and mental health issues arising from this post-industrial lifestyle creep, and respond to these evolving global challenges by calling for equitable access to unstructured play in natural environments. In comparison to their 2015 Position Statement on Active Outdoor Play which addressed Canadian youth, the updated version has expanded the scope of its recommendations to now encompass “all age groups” and “a global perspective”.

Reflecting this pressing global need for accessible lifestyle changes, bonded green exercise is hypothesized to offer a biologically plausible, emotionally resonant, and low-cost behaviour that plausibly reconnects humans and dogs with their evolutionary roots of shared movement, social cooperation, and environmental immersion. Unlike prescriptive health advice that is often costly and targets individual deficits (e.g., move more, or spend time in nature, or connect with others), bonded green exercise integrates all three domains at once, thereby positioning it as a potentially uniquely powerful and testable behavioural model for addressing modern health challenges in both species. Because bonded green exercise is inherently low-cost and, we posit, likely motivating for many people—as supported by related studies examining various constituent elements [13,49,55,58,94]—it also supports our hypotheses H3 (behavioural amplification) and H4 (scalable health promotion).

## 4. Existing Evidence Across the Three Core Domains

The following subsections are not intended to be an exhaustive review of the literature, but rather to synthesize key evidence of health outcomes across the domains.

### 4.1. Nature Exposure

Exposure to natural settings affords mental and physical benefits to both humans (Table 1) and dogs (Table 2). For humans, the body of evidence is well-established and has been studied at various scales [95]; for dogs it is an emerging area of research, often in conjunction with studies on HAI and behavioural enrichment.

**Table 1:** Selected health outcomes of nature exposure in humans.

Category	Outcomes	Supporting References
Stress & autonomic regulation	Reduced stress, lower cortisol levels, decreased sympathetic activity, improved mood, improved mental health, & emotional regulation	[40,96]
Cardiovascular health	Lower blood pressure, healthier cardiovascular profiles	[97]
Immune function	Improved immune function	[97]
Psychological, cognitive & neurological function	Cognitive restoration, enhancing attentional capacity, working memory, overall cognitive performance; increased neuroplasticity & proprioceptive feedback, which helps preserve balance & mitigate neurological decline	[98,99]
Well-being & longevity	Higher life satisfaction, greater overall well-being, reduced mortality	[97,100]

**Table 2:** Selected health outcomes related to nature exposure in dogs.

Category	Outcomes	Supporting References
Neurological function	Heightened proprioceptive feedback, which helps preserve balance & mitigate neurological decline	[101]
Musculoskeletal function	Engaging diverse muscle groups through varied terrain	[41,101]
Sensory enrichment	Exposure to biodiverse odours & volatile organic compounds, neurophysiological enrichment	[66,102]
Microbiome health	Increased gut microbiota diversity	[103]
Well-being, agency, & affective state	Increased opportunity for expressing natural behaviours (e.g., digging; off-leash autonomy) promoting healthier stress profiles, greater confidence, a stronger human–animal bond	[15,22,31]

#### 4.1.1. Nature Exposure Health Outcomes in Humans

In humans, evidence from epidemiological and population-based studies links exposure to natural environments to benefits across multiple physiological and psychological systems (Table 1).

A study from the U.K. Biobank identified that adults living in greener neighbourhoods have lower mortality rates, even after accounting for socioeconomic and demographic factors. Mediation analyses suggest these benefits may operate through reduced air pollution exposure, improved mental health, and decreased social isolation [100].

Emerging evidence suggests that aquatic environments (‘blue space’) provide comparable mental-health benefits; a review by White et al. [104] highlighted improvements in mental health and prosocial effects from exposure to blue space.

Importantly, these outcomes appear across a wide range of natural settings—from small urban parks and tree-lined streets to larger natural areas—indicating that meaningful health benefits do not depend on pristine wilderness but on the presence and accessibility of nature itself [105]. Current guidance recommends 120 min per week of nature exposure to achieve measurable benefits [104].

#### 4.1.2. Nature Exposure Health Outcomes in Dogs

Spending time in natural settings provides dogs with mental and physical enrichment largely lacking when walking on sidewalks amongst concrete buildings. As shown in Table 2, empirical studies reveal that exposing dogs to natural environments provides the potential for multiple body systems to benefit, including proprioceptive, musculoskeletal, sensory, and agency-linked processes.

Natural settings also afford welfare advantages, with opportunities for dogs to display autonomy and engage in exploratory behaviours and decision-making, such as being allowed to choose the pace and/or which trail to take.

Within the framework of bonded green exercise, nature-based interventions, if experienced together by a human and dog, may plausibly strengthen the preconditions for cross-species benefits (H2).

### 4.2. Physical Activity

The long-recognized principle that “motion is medicine”, a modern adaptation of Hippocrates’ assertion that walking is “man’s best medicine” [106] (p.1) has become a guiding mantra among health professionals for good reason. This principle applies equally to canines. Physical activity engages and benefits nearly every physiological and neural system, promoting metabolic efficiency, cardiovascular health, musculoskeletal integrity, neuroplasticity, and emotional stability (Table 3).

**Table 3:** Selected health outcomes of physical activity in humans.

Category	Outcomes	Supporting References
Stress & autonomic regulation	Improved stress and emotional regulation; reduced anxiety	[107,108]
Cardiovascular health	Improved circulation, oxygenation, & cellular repair; improved cardiovascular health	[109,110,111]
Musculoskeletal function	Preserved lean body mass; improved bone density, flexibility, joint mobility	[111]
Metabolic health	Increased insulin sensitivity, reduced adiposity	[112]
Psychological, cognitive & neurological function	Optimal cognitive function & mental well-being; increased neuroplasticity, executive function, & memory; reduced age-related cognitive decline; reduced rates of depression & anxiety	[108,109,113]
Well-being & lifespan	Enhanced longevity; reduced risk of chronic non-communicable diseases; reduced all-cause mortality	[109,110,111]

#### 4.2.1. Physical Activity Health Outcomes in Humans

Across populations, physical activity is among the most well-studied determinants of health and longevity. Global recommendations from the World Health Organization [114], the Physical Activity Guidelines for Americans [115], and the Canadian 24-Hour Movement Guidelines [116] advise a minimum of 150 min of moderate-to-vigorous activity per week for adults. Yet worldwide, more than a quarter of adults and four-fifths of adolescents fail to meet these targets [117]. Physical inactivity is estimated to account for roughly 9% of premature mortality [109].

Physical activity confers wide-ranging benefits across human physical and psychological systems (Table 3). Framing bonded green exercise within a public-health context underscores its potential as a motivating way to meet and sustain recommended physical activity levels, while potentially improving human well-being and longevity.

#### 4.2.2. Physical Activity Health Outcomes in Dogs

Regular physical activity is paramount to a dog’s health and overall well-being [101], particularly when giving them an opportunity for agency through off-leash exploration [37], yet only 30–60% of dog owners walk their dogs regularly [48,118]. Exercise, especially that which improves core strength, contributes to reduced risk of musculoskeletal injury [101,119,120], reduced body condition score, and improved lean body weight [41]. Physical activity also increases mobility and joint health, and contributes to improved balance and proprioception, especially in dogs with osteoarthritis and in senior dogs. Yet, over 50% of dogs in North America are overweight or obese [14], a debilitating condition associated with elevated risk of chronic metabolic diseases, orthopedic conditions, and reduced lifespan [8,9].

### 4.3. Human–Animal Bond

The human–animal bond is distinct in its depth and reciprocity, particularly within an individual human–dog dyad [24]. Unlike most interspecies relationships, humans and dogs are capable of an attachment bond akin to that typically observed between conspecific sexual partners or parents and their offspring [24,32,80,121]. These physiological and emotional effects are not unidirectional: mutual interactions such as eye contact, touch, and shared activity trigger bidirectional changes in both species, reinforcing the bond.

The attachment bond is related to the oxytocin (OT) positive-feedback loop, whereby OT release occurs in humans and dogs in response to positive interactions and social cues. In turn, OT strengthens the bond, enhances trust, reduces cortisol levels, and buffers stress in both species. These attachment dynamics include proximity seeking, secure base effect, and stress buffering—features that are emotionally and physiologically beneficial for both members of the dyad.

#### 4.3.1. Bond-Related Health Outcomes in Humans

For humans, engagement with a bonded dog has been associated with a wide range of biopsychosocial benefits (Table 4).

**Table 4:** Selected health outcomes of the human–animal bond in humans.

Category	Outcomes	Supporting References
Stress & autonomic regulation	Reduced stress reactivity & cortisol levels; enhanced oxytocin release; improved emotional regulation; suppressed hypothalamic–pituitary–adrenal (HPA) axis activation; elevated heart rate variability	[43,44,45,80,122,123]
Attachment bond & oxytocin pathway activation	Enhanced oxytocin release during affiliative interactions (e.g., touching, gaze, shared activity) increases bonding, trust, cooperation, social reward	[80,123]
Cardiovascular health	Lower blood pressure, elevated heart rate variability, & overall cardiovascular resilience	[45,87]
Psychological, cognitive & neurological function	Increased prosocial behaviour & empathy; social stress modulation; decreased loneliness, companionship; increased cognitive function	[30,32,48,49,53,54,60,123,124,125,126,127,128]
Well-being, social function & motivation	Greater participation in outdoor exercise and social activities across age groups; increased life satisfaction	[49,50,51,126,127,128,129,130,131]

These outcomes are consistent with the broader literature on social capital and “togetherness” [52], suggesting that the HAB functions as a form of interspecific social support that complements human–human networks.

Importantly, the motivational effects of attachment, particularly a sense of responsibility and emotional reciprocity toward the dog, may strengthen adherence to health-promoting behaviours such as regular walking or outdoor recreation [49,50,51,126,129,130]. These aspects that motivate people to be physically active have been observed across all ages including children [49] and the elderly [127,128,131] and are correlated with the strength of the bond between the human and the dog. Owners who feel a secure, emotionally rewarding bond with their dog may be more likely to engage in consistent shared activity, underscoring the relational drivers of outdoor physical activity.

In this way, the human–animal bond not only appears to enhance well-being directly through affective and physiological mechanisms but also indirectly by plausibly catalyzing sustained engagement in shared physical activity and nature exposure. This hypothesized behavioural amplification effect (H3) plausibly situates the human–dog bond as both a relational and a practical driver of improved health outcomes.

#### 4.3.2. Bond-Related Health Outcomes in Dogs

In dogs, the human–animal bond supports physiological, emotional, and welfare outcomes (Table 5).

**Table 5:** Selected health outcomes of the human–animal bond in dogs.

Category	Outcomes	Supporting References
Stress & autonomic regulation	Reduced cortisol levels; enhanced oxytocin release; suppressed hypothalamic–pituitary–adrenal (HPA) axis activation; stress buffering; elevated heart rate variability	[43,44,45,57,79,80,122,132,133]
Attachment bond & affiliative behaviour	Enhanced oxytocin release; increased bonding, trust, cooperation, social reward; secure attachment behaviours	[32,43,46,80]
Affective well-being & healthspan	Improved mental well-being, curiosity, engagement & exploration	[15,22,32,46,57]

The magnitude of these benefits appears to correlate with the owner’s attachment style [134] and the strength of the attachment bond, the latter which is amplified by time spent together, particularly when that time involves mutual engagement. Even shelter dogs, who typically have weaker attachments to their human caregivers than pet dogs, demonstrate measurable physiological and behavioural outcomes from human interaction, though to a lesser extent than in established human–dog dyads [57,75,132,133,135]. This gradient of response suggests that while the effects of human interaction are broadly beneficial, they are most pronounced when the relationship involves consistent, reciprocal engagement. In other words, it is not only the amount of time spent together but the quality of that interaction that determines the depth and biological impact of the attachment.

This gradation of physiological and behavioural attunement across relationship types underscores that the HAB operates along a continuum in both species, where increasing familiarity and reciprocity may plausibly amplify the regulatory and affiliative effects most relevant to the bonded green exercise framework.

The relevance of the HAB for bonded green exercise lies in its hypothesized potential (H1) to amplify the benefits of nature exposure and physical activity. The aforementioned evidence lends support to our hypotheses that when humans and dogs intentionally engage together in outdoor environments, their bond is not only potentially expressed but we posit that it may plausibly be actively strengthened through mechanisms such as oxytocin-mediated co-regulation, opportunities for agency, and reciprocal trust. Synchrony of movement and affect, though still an emerging area of research, has been found to co-occur with these processes and therefore we posit that these processes in human–dog dyads may contribute to reinforcing their attachment bond over time.

### 4.4. One Health Perspective

The One Health framework emphasizes the interdependence of human, animal, and environmental health [38]. Bonded green exercise exemplifies this perspective by potentially unlocking health benefits across all three domains simultaneously. For humans, shared outdoor activity supports physical fitness, stress reduction, trust, mental and social well-being. For dogs, it plausibly enhances welfare through exercise, mental enrichment, agency, trust, stress reduction, and secure attachment. For the environment, increased use and valuing of green and blue spaces may foster stewardship and awareness of ecological systems. By situating bonded green exercise within the One Health paradigm, we highlight its relevance not only as an individual-level behaviour but also as a potentially population-level intervention that connects canine science, public health, and an appreciation for environmental sustainability.

## 5. Existing Evidence on Domain Pairings

There is a collective abundance of studies on the health benefits, primarily in humans, of experiencing two of these domains concurrently:

### 5.1. Nature Exposure + Physical Activity (Green Exercise)

The synergistic effects of green exercise on mental and physical health in humans has been well documented [136]. These outcomes include elevated self-esteem and mood (determinants of mental health) over exercise without nature [59], as well as greater feelings of revitalization and energy when exercising outdoors compared to indoors [58].

Positive effects on mood and self-esteem from green exercise can emerge rapidly, even after brief exposure to an urban or rural natural setting [84]. Collectively, these findings demonstrate that even short, accessible interactions with nature can produce measurable improvements in mood and well-being, underscoring the scalability of nature-based interventions in urban contexts.

In addition to the public health benefits of green exercise, being physically active in nature encourages a mindset of environmental awareness and stewardship, consistent with One Heath and with the urgent need for conservation efforts for our planet.

While the established benefits of green exercise support that combining nature and physical activity may yield outcomes greater than those domains alone, the dimension of connection—a core determinant of emotional and physiological well-being in humans and dogs (as social mammals)—has not been explicitly examined within the context of green exercise.

Incorporating the human–animal bond introduces a relational variable that may plausibly further amplify motivation (H3), reward (H3), and physiological co-regulation. In this way, bonded green exercise extends the green-exercise paradigm from an environmental-behavioural model to a conceptualized environmental-behavioural-relational one, which we hypothesize may unlock a synergistic benefit for both species (H1 and H2).

### 5.2. Physical Activity + HAB

Engaging in physical activity with dogs may benefit both members of the dyad. Dog ownership is associated with higher levels of physical activity across age groups in humans [137,138] and across age groups and breeds of dogs [139,140]. Dog-associated physical activity increases the likelihood of meeting public-health exercise guidelines [48,50,129] and is linked to improved mental health and life satisfaction [131]. Among children and adolescents, stronger attachment to the family dog predicts higher levels of outdoor play and leisure-time activity [49]. Regular walking and play also support canine weight management, musculoskeletal conditioning, and behavioural well-being [41,101,129].

Beyond compliance with activity targets, physical activity involving dogs leverages relational and emotional motivation. This motivational reciprocity has the potential to transform exercise from a solitary discipline into a shared, emotionally reinforcing behaviour, thus supporting bonded green exercise’s behavioural-amplification hypothesis (H3).

However, despite the extensive research on dog ownership and physical activity, relatively few studies have investigated how these behaviours intersect with psychosocial outcomes such as loneliness and mental health [60].

Given the reported amplified effects of green exercise [58,59,98,141], we hypothesize that the inclusion of the human–animal bond may plausibly further amplify outcomes. Specifically, relational engagement may potentially influence how physical activity is initiated, sustained, and emotionally experienced, thereby plausibly enhancing the effects of physical activity rather than simply adding benefits. As dog-facilitated activity has been shown to improve exercise motivation, adherence and affective reward, we posit that embedding that behaviour in natural environments may enhance both physiological and emotional gains for human and dog alike. Existing evidence from dyadic (human + dog) and environmental (nature + exercise) research together lend conceptual plausibility to our triadic-synergy hypothesis (H1) of bonded green exercise.

### 5.3. Nature Exposure + HAB

The biophilia hypothesis [42,82] proposes that humans possess an evolved, innate affinity for life and life-like processes, including animals and nature. This affinity is thought to confer adaptive advantages by directing attention and emotional attachment toward natural environments and living beings that supported survival across evolutionary time. Within the context of bonded green exercise, biophilia provides a unifying theoretical mechanism linking the attraction to nature with the attraction to animals, particularly dogs. That is, plausibly providing the deep biological wiring for a powerful bond, while we posit that culture and individual agency may provide the opportunity to re-activate it.

Current evidence, as aforementioned, supports that both exposure to nature and affiliative human–animal contact may plausibly activate overlapping regulatory systems related to stress modulation and reward processing. Affiliative contact with animals has been empirically linked to oxytocin release, and nature exposure has been hypothesized to involve activation of the oxytocin pathway as well (e.g., [142]). We hypothesize that when these experiences co-occur (e.g., engaging with one’s dog in a natural environment) the resulting experience may plausibly amplify positive affect, attention restoration, and physiological co-regulation--potentially beyond the effects of either domain alone. This line of reasoning illustrates the conceptual basis for our broader hypothesis (H1) that bonded green exercise may plausibly yield synergistic benefits in part by simultaneously engaging multiple systems.

Having co-evolved alongside humans within shared ecological contexts, the presence of a human partner may further shape how dogs perceive and engage with natural environments. For dogs, exploration of natural environments in the presence of a trusted human partner may be interpreted through a social lens that promotes curiosity, confidence, and engagement rather than vigilance or uncertainty (see Table 5, Affective well-being & healthspan).

As such, dogs are more likely to interpret novel situations positively and to explore the environment when their human partner is present and offers positive cues [32,46,73,87]. When an animal perceives safety (such as being in the presence of a positive attachment figure), sympathetic tone decreases while motivation for seeking and play behaviours is engaged [143]—both core components of well-being. We argue that this mechanism may likely be at play during active exploration in natural settings with a bonded human, where relational security and affiliative cues promote physiological calm and curiosity-driven engagement. Nature exploration with a human partner may hence plausibly enhance a dog’s engagement, enrichment, and well-being during shared outdoor activity (H2).

Collectively, this body of evidence illustrates the health benefits of individual domains as well as domain pairings, and provides a conceptual basis for our untested hypotheses of triadic synergy (H1) to both species (H2) as well as potential for bonded green exercise to be inherently motivating (H3) and scalable (H4). Existing studies also underscore the gap: to the authors’ knowledge, no framework yet examines the effect of all three domains in full integration, and on both species. This gap in empirical evidence provides the conceptual rationale for the bonded green exercise framework proposed below.

## 6. Proposed Framework for Bonded Green Exercise

**Figure 1:**
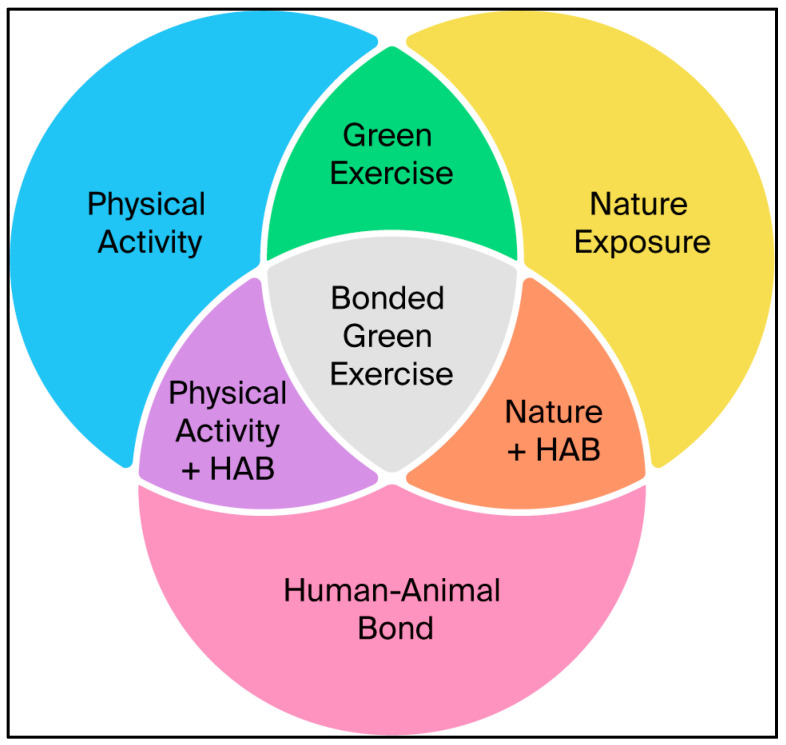
Conceptual framework of bonded green exercise: Venn diagram showing the three domains included in the bonded green exercise framework: Nature Exposure, Physical Activity, and the Human–Animal Bond (HAB). Overlapping regions indicate combined domains, with the central overlap representing bonded green exercise.

Bonded green exercise builds upon the preceding evidence of health outcomes by conceptualizing nature exposure, physical activity, and the human–animal bond as three interdependent domains that may plausibly converge to produce synergistic outcomes for both species (Figure 1), through overlapping physiological, behavioural, and affective mechanisms.

### 6.1. Biobehavioural Mechanisms Plausibly Engaged During Bonded Green Exercise (H1 and H2)

Rather than treating nature, movement, and relationship as discrete variables, the bonded green exercise framework positions their intersection as a plausible physiological and behavioural system. We posit that this triadic combination of domains may yield a dynamic state in which shared activity between human and dog plausibly activates conserved mechanisms of regulation and reward that may be unlikely to arise in isolation.

Drawing on aforementioned existing evidence from the three domains, we suggest five plausible and testable mechanisms which may be present during bonded green exercise:

- *Physiological co-regulation*: Alignment of stress- and autonomic-related markers between dogs and humans during mutual engagement, particularly when attachment bonds are strong.
- *Neural synchrony*: Interbrain neural coupling from human to dog during mutual attention and affiliative interaction.
- *Behavioural synchrony*: Spontaneous coordination of gait, pacing, and attention during shared movement, fostering attunement and trust.
- *Affective reinforcement*: Reciprocal emotional buffering and shared reward that enhance positive affect, deepening of the attachment bond, and motivation to repeat the behaviour.
- *Agency-supported bonding*: Natural environments allow dogs to exercise choice and exploratory behaviour, deepening trust and attachment while promoting human responsiveness.

These processes, if empirically determined to be engaged during bonded green exercise, lend support to our hypothesis that bonded green exercise may plausibly function both as a lifestyle practice and as a biobehavioural framework supporting mutual regulation and well-being of the dyad (Table 6).

**Table 6:** Hypothesized mechanisms and plausible corresponding health benefits supporting the bonded green exercise conceptual framework of shared physical activity in nature of human–dog dyads.

Mechanism	Outcomes	Supporting References
Physiological coregulation	Oxytocin elevation; reduced cortisol alignment of heart rate variability between human and dog; parasympathetic activation; mutual engagement	[15,32,43,44,45,53,57,79,80,122,132,144,145]
Neural synchrony	Interbrain neural coupling	[146]
Behavioural synchrony	Mirroring of gate, pace, & posture; mutual gaze coordination; improved attunement & trust	[75,76,135,147]
Affective reinforcement	Reciprocal, emotional buffering; increased positive affect; strengthened attachment bond & motivation to repeat activity	[22,32,49,53,55,57,86,124,148,149]
Agency-supported enrichment and bonding	Enhanced canine agency & exploratory behaviour; improved trust & attachment bond from joint exploration	[15,22,37,46,47,53,87,150,151]

As this bonded green framework remains provisional, we propose the following methods for how the above four mechanisms may plausibly interconnect (H1 and H2) to produce mutually synergistic and self-reinforcing outcomes in the human–dog dyad:

- *Physiological co-regulation ↔ Affective reinforcement*: Emotional reciprocity both drives and results from physiological alignment. The more relaxed and bonded each partner feels, the more their autonomic states synchronize, and that synchrony further deepens positive emotion and trust.
- *Behavioural synchrony ↔ Physiological co-regulation*: When gait, pace, and gaze are aligned, this shared movement directly supports trust, parasympathetic activation and stress reduction. Conversely, physiological calm and attunement make behavioural synchrony easier to sustain.
- *Affective reinforcement ↔ Behavioural synchrony*: Positive emotional feedback increases the likelihood of continued synchronous movement, thus producing a rewarding emotional state that further reinforces the attuned behaviour.
- *Agency-supported enrichment ↔ Affective reinforcement*: When dogs experience choice and curiosity, their sense of safety and exploration increases positive affect; that shared affective state strengthens the bond and motivation to engage again.
- *Agency-supported enrichment ↔ Physiological co-regulation*: A relaxed, safe physiological state allows exploratory behaviour; conversely, exploration in a supportive context reinforces calm through predictability and trust.
- *Agency-supported enrichment ↔ Behavioural synchrony*: Shared exploration often includes parallel attention and coordinated movement patterns; synchrony helps the dog feel safe to exercise agency, and that agency enhances synchrony by deepening trust and mutual awareness.

**Figure 2:**
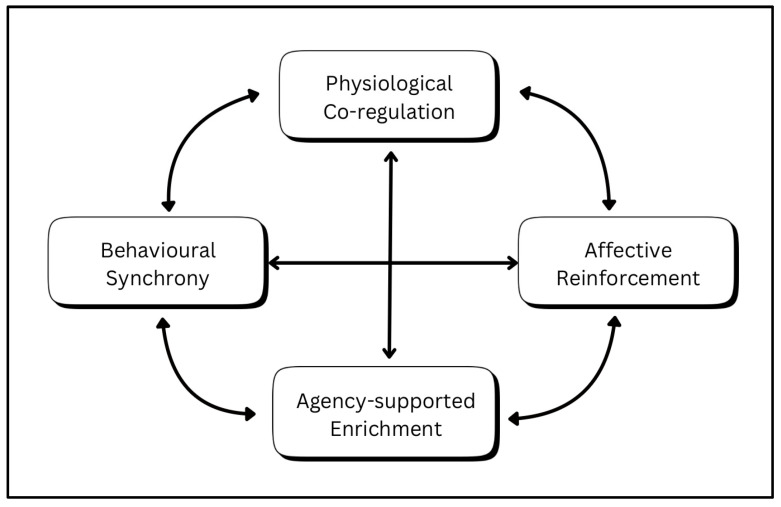
Conceptual diagram illustrating four components hypothesized to plausibly bidirectionally interact in the human–dog dyad within the provisional framework of bonded green exercise: physiological co-regulation, behavioural synchrony, affective reinforcement, and agency-supported enrichment.

The potential interconnectedness of these processes (Figure 2) suggests that bonded green exercise may plausibly generate outcomes greater than the sum of its parts (H1), with benefits in one partner reciprocally amplifying those in the other (H2).

### 6.2. The Dog as a Catalyst

Within this framework, we argue that the dog may additionally function as a motivational catalyst. As a social partner, the dog has been shown to enhance human adherence to physical activity and time spent outdoors (see Table 4, Well-being, social function & motivation), while the human facilitates the dog’s access to enriched, autonomy-supportive contexts (see Table 5, Affective well-being & healthspan). This mutual influence strengthens the bond, and we posit that this may create a self-reinforcing feedback loop in which affective reward and behavioural consistency plausibly amplify each other. Such motivational reciprocity has been observed across age groups and living contexts, lending support to our behavioural amplification hypothesis (H3).

### 6.3. Scalability of Bonded Green Exercise

The ubiquity of dog ownership combined with the low cost and accessibility of shared outdoor activity render bonded green exercise plausibly scalable across diverse populations, lifestyles, and environments (H4). In essence, bonded green exercise provides a model to plausibly enhance physical and mental health, connection, and ecological engagement simultaneously across species.

As a testable conceptual framework, bonded green exercise may plausibly further serve as a template for broader relational models of green exercise, potentially extending to other interspecies or human–human partnerships (such as between human couples, or between a horse and a horseback rider) where other forms of synchronized movement and mutual regulation occur within natural environments.

## 7. Limitations and Future Research Directions

Bonded green exercise is proposed here as a provisional conceptual framework with testable hypotheses, rather than a prescriptive intervention or methodological protocol. Accordingly, the considerations below delineate conditions and moderating factors for empirical investigation, rather than specifying standardized methods or outcomes. As with any heterospecific model, its application and evaluation must account for contextual, relational, and biological variability.

### 7.1. Moderating Factors Within the Human–Dog Dyad

The effects of bonded green exercise are expected to be moderated by characteristics of the human–dog relationship itself. Outcomes may be influenced by factors such as bond quality and duration, attachment style, canine temperament, and breed-linked traits, as well as by the human’s attentiveness and responsiveness during shared activity. For instance, an owner’s personality and attachment style (e.g., secure vs. avoidant) have been shown to affect the strength of the bond as well as the behaviour and anxiety levels of their dog [22,152]. Similarly, some dog breeds (e.g., Shepherds) are inherently more likely to be human-focused during shared activity—and this may affect their level of engagement with their owner [147]. While these factors are plausibly expected to shape the *magnitude and expression* of observed effects, bonded green exercise is still hypothesized to confer *net positive* outcomes to the dyad, based on the aforementioned mechanisms including the strengthening of the bond through intentional shared activity.

Physical characteristics and conditioning of both partners also warrant consideration. The type, intensity, and duration of shared activity should be appropriate to the dog’s age, breed, health status, and physical capacity, as well as to the human’s abilities. As bonded green exercise is adaptive and context-dependent, inherent heterogeneity across dyads should therefore be understood not as a limitation of the framework, but as a feature that shapes its expression.

### 7.2. Ethical and Welfare Considerations

Shared human–dog activity in nature requires responsible management to ensure the welfare of the dog, the human, and the surrounding environment. Considerations include physical limitations, as well as preventing overexertion or overheating of the dog, mitigating stress or fear responses, complying with leash regulations, practicing “leave no trace” principles, and avoiding disruption to wildlife and ecological systems.

These responsibilities are not unique to bonded green exercise but reflect best practices inherent to any outdoor engagement with companion animals, and are essential considerations for ethical public health applications, particularly when welfare-supportive activities are intentionally promoted.

While such considerations necessarily focus on preventing adverse outcomes, contemporary welfare frameworks emphasize that positive states—including agency, engagement, positive affect, and a strong human–animal bond—are equally essential components of a dog’s well-being [153,154], and we posit that such states may be supported through appropriately designed bonded green exercise.

### 7.3. Contextual and Accessibility Limitations

Access to suitable green space and to canine companionship is unevenly distributed across geographic, socioeconomic, and cultural settings. Although large natural landscapes may not be universally available, aforementioned evidence suggests that even modest urban green spaces—such as neighbourhood parks, community gardens, or tree-lined walking paths—can elicit measurable physiological and psychological benefits. This supports the relevance of bonded green exercise within urban environments, where opportunities for nature exposure and physical activity may otherwise be limited and highlights its potential applicability within population-level One Health initiatives.

The emphasis on low-cost, accessible, and everyday contexts suggests that the bonded green exercise framework may be compatible with population-level public health promotion and One Health initiatives, without requiring specialized infrastructure.

Having said this, bonded green exercise is also not universally applicable. Across cultures and individuals, not every human wishes to own or interact with dogs, and not all dogs are suited to shared outdoor activity in all settings. For individuals without access to a companion dog, community-based initiatives such as shelter-dog walking or hiking programmes may offer partial opportunities for shared outdoor activity. While such relationships are typically less established than long-term cohabitating dyads, existing evidence suggests they may still confer physiological and affective benefits for both species [57,132].

### 7.4. Empirical Testing and Methodological Considerations

As a conceptual framework, bonded green exercise is intended to guide, rather than prescribe, empirical investigation. Researchers across disciplines may employ diverse methodological approaches reflecting differences in research questions, study populations, and measurement advancements.

Conceptually, empirical studies on bonded green exercise may wish to compare conditions in which all three domains are present versus conditions in which one or more components are selectively absent. For example, within-subject or crossover designs could compare shared human–dog activity in nature with paired conditions in which one domain is absent (e.g., green exercise with versus without a dog; dog-walking while being ‘present’ versus while being ‘distracted’ on one’s smartphone; or human–dog interaction in nature with versus without physical activity). This framing offers a simple organizing heuristic—shared, active, and green—through which the hypothesized effects of bonded green exercise may be isolated and tested.

Across such designs, outcomes of interest may include physiological indicators (e.g., stress regulation or autonomic balance), behavioural outcomes (e.g., activity synchrony; social referencing), affective or psychological measures in both humans and dogs, and subjective attachment scores.

The following provides illustrative criteria through which the four hypotheses presented here may be empirically investigated:

Hypothesis 1 (triadic synergy) would be supported if shared activity in nature produces effects to one or both members of the human–dog dyad that are greater in magnitude, more persistent, or qualitatively distinct compared to those observed when physical activity, nature exposure, or human–dog interaction are experienced in isolation. Conversely, equivalent or lesser effects would constitute disconfirming evidence and help to refine the framework’s scope.

Hypothesis 2 (parallel benefits) may be examined through concurrent assessment of physiological, behavioural, or affective outcomes in both members of the human–dog dyad, such as cortisol or heart rate variability measurements, synchrony of activity patterns, or shared affective responses.

Hypothesis 3 (behaviour amplification) may be evaluated through indicators of motivation, adherence, and affective reward, including increased frequency or duration of shared activity, sustained engagement over time, or self-reported and observed enjoyment relative to non-bonded or non-natural activity contexts.

Hypothesis 4 (scalability and accessibility) may be informed by studies conducted across diverse environmental settings, demographic groups, and dyad characteristics, including urban green spaces and varied relationship contexts, without requiring ‘backcountry’ natural environments or specialized physical activities. For example, bonded green exercise may be examined within everyday human–dog routines by studying dyads engaging in intentional, shared activity within locally accessible green spaces, such as an intentionally shared walk in a neighbourhood park, thereby allowing investigation of scalability under conditions that are widely available and minimally resource-intensive.

Reported outcomes across HAB/HAI research remain inconsistent, most likely reflecting differences in study design, measurement, and context [53,125,155,156]. Additionally, differences in breed attributes, such as the inherent level of human-directed attention, should be taken into account during study design. In Martin et al. [22], the authors provide a discussion of limitations of, and recent advancements in, HAI methodology. Furthermore, the recent availability of activity trackers for humans and pets mitigates motion artefacts of physiological measurements during free-moving activities [146,148,157].

Future investigations of bonded green exercise should therefore prioritize standardized constructs and outcome measures to strengthen reproducibility and cross-study coherence [155], as well as implement best-practices of canine welfare to mitigate stress levels and enhance well-being [143,158]. By integrating these methodological insights, bonded green exercise research may also contribute to improving methodological rigour within HAB and HAI science.

## 8. Conclusions

In this manuscript we discussed the parallel health costs to humans and dogs of industrialized society’s screen-addicted, sedentary, indoor lifestyle, and introduced the first conceptual framework that integrates nature exposure, physical activity, and the human–animal bond into a single model of shared human–dog activity—bonded green exercise. Importantly, we posited that this testable framework may plausibly confer meaningful health outcomes through concurrent, additive effects of these three well-studied domains, independent of any hypothesized synergistic amplification.

We proposed that this One Health conceptual framework may have the potential to enhance health and lifespan across species. We hypothesized the plausible physiological and behavioural underpinnings of synergistic benefits to dogs and humans, as well as how dogs may potentially act as a catalyst, motivating their humans to be active together outdoors. Finally, we discussed how bonded green exercise is potentially low-cost, widely accessible, and scalable to enhance public and canine health in industrialized societies worldwide.

Grounded in their shared evolutionary history and consistent with One Health principles, we suggested that bonded green exercise may plausibly represent a biologically coherent and accessible behaviour that may potentially improve human and canine physical, emotional, and relational well-being, including fostering the human–animal bond. Furthermore, since canine welfare depends not only on physical health but also on environmental quality, expression of natural behavioural, opportunities for agency, positive affective experiences, and the quality of the human–animal bond [153,154], bonded green exercise may plausibly provide a practical and scalable pathway for improving everyday welfare in companion dogs.

Overall, this novel provisional framework may offer potential for broad-reaching implications for future public health policies and One Health practice. This testable framework and its hypotheses invite empirical research and interdisciplinary studies exploring how shared nature-based physical activity may promote the health, welfare, and relational well-being of both humans and dogs.

## Data Availability

No new data were analyzed or created during this study. Data sharing is not applicable.

## Abbreviations

The following abbreviations are used in this manuscript:

HAB	Human–animal bond
HAI	Human–animal interaction
WHO	World Health Organization
